# Improving the Accuracy of a Wearable Uroflowmeter for Incontinence Monitoring Under Dynamic Conditions: Leveraging Machine Learning Methods

**DOI:** 10.3390/bios15050306

**Published:** 2025-05-11

**Authors:** Faezeh Shanehsazzadeh, John O. L. DeLancey, James A. Ashton-Miller

**Affiliations:** 1 Department of Mechanical Engineering, University of Michigan, Ann Arbor, MI 48109, USA; jaam@umich.edu; 2 Department of Obstetrics & Gynecology, University of Michigan, Ann Arbor, MI 48109, USA; delancey@med.umich.edu

**Keywords:** uroflowmeter, urinary incontinence, wearable device, dynamic flow conditions, flow conditioner, machine learning, extreme gradient boosting, support vector machines

## Abstract

Urinary incontinence affects many women, yet there are no monitoring devices capable of accurately capturing flow dynamics during everyday activities. Building on our initial development of a wearable personal uroflowmeter, this study enhances the device’s performance under realistic, dynamic conditions similar to those encountered in daily living. We integrated an optimized eight-vane Etoile flow conditioner with a 0.2D opening into the device. Both computational fluid dynamics simulations and experimental tests demonstrated that this flow conditioner significantly reduced turbulence intensity by 82% and stabilized the axial velocity profile by 67%, increasing the R^2^ of flow rate measurements from 0.44 to 0.92. Furthermore, our machine learning framework—utilizing a support vector machine (SVM) and an extreme gradient boosting (XGBoost) model with principal component analysis (PCA)—accurately predicted the true flow rate with high correlations, robust performance, and minimal overfitting. For the test dataset, the SVM achieved a correlation of 0.86, an R^2^ of 0.74, and an MAE of 2.8, whereas the XGBoost-PCA model exhibited slightly stronger performance, with a correlation of 0.88, an R^2^ of 0.76, and an MAE of 2.6. These advances established a solid foundation for developing a reliable, wearable uroflowmeter capable of effectively monitoring urinary incontinence in real-world settings.

## 1. Introduction

Urinary incontinence (UI), the involuntary loss of urine, affects approximately 200 million people worldwide, with up to 55% of women experiencing it at some point in their lives [1,2,3]. The prevalence of UI increases with age, affecting 17% of women over 20 years old and up to 77% of elderly women [2,4,5]. Additionally, 37.5% of young women (ages 30–50) in primary care settings report stress incontinence [6]. Stress urinary incontinence (SUI), a common subtype, is characterized by involuntary leakage during increases in abdominal pressure associated with jump landings, lifting, sneezing, and coughing [1,2]. Despite its prevalence, UI remains underdiagnosed and undertreated, with only 25% of affected women seeking care [2]. This condition significantly impacts quality of life, leading to psychological distress and greater burdens on health resources [4,7], yet the causes of UI in adult women are not fully understood [8].

Diagnosis typically requires a comprehensive evaluation involving detailed history-taking, physical exams, and urodynamic testing [2,4]. It is crucial to determine the severity of symptoms to tailor treatment effectively [1]. This relies on closely monitoring the frequency and volume of urine leakage to identify triggers and underlying issues. This approach allows healthcare providers to refine treatment strategies, offering personalized interventions that significantly enhance patient quality of life [9,10]. While urodynamic tests are considered the gold standard for assessing bladder and urethral function and identifying urinary incontinence subtypes, their invasive nature and high costs limit widespread application [2,7,11,12]. Clinicians often use validated questionnaires and leakage diaries to track symptoms [13], although these methods can be impractical because patients do not always notice or remember leakage episodes, with recall bias particularly affecting older adult data [1,2,14,15,16]. Pad tests quantify urine loss by measuring the weight gain of absorbents over a fixed time period (e.g., 24 h), but do not capture real-time flow rates [1,13,17]. Alternatives such as wetness sensor wearables [18,19,20], urine collection bags [17], and the paper towel test [21] are available but fail to measure instantaneous flow rates or the number of leakage episodes during activities of daily living (ADL).

Uroflowmetry measures urine flow rates and voiding time, typically in a seated posture on a commode, but requires specialized lab equipment and settings, obviating its application in monitoring daily incontinence [22,23]. Recent advances aim to improve flexibility through portable and handheld devices, but these face challenges of inaccuracy and sensitivity to movement and urine salinity [22,23]. Although home-based monitoring solutions present potential advantages, they are often limited by complexity and cost, emphasizing the need for accessible, reliable devices for everyday use [24,25,26].

To address these issues, we designed a novel wearable personal uroflowmeter (PUF) for monitoring SUI during ADL [27,28]. The PUF attaches over the urethral meatus in women, measuring urine flow rates and volume during ADL. It also incorporates a waist-mounted inertial measurement unit (IMU) to capture physical activity and posture in real-time to enable post hoc identification of which ADL triggered a leakage episode, along with the amount of leakage. This article describes enhancements to the PUF design and signal processing in order to achieve precise incontinence monitoring under dynamic conditions by utilizing machine learning (ML) techniques.

**Figure 1:**
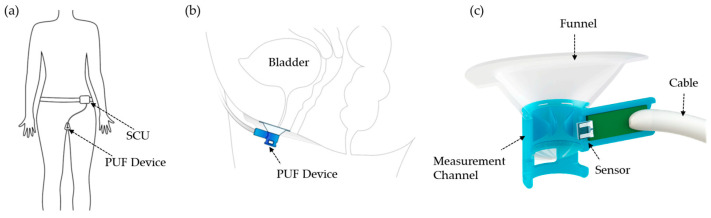
(**a**) Front view showing apparatus worn by the subject, (**b**) Left lateral schematic showing the PUF placement over the female urethral meatus, (**c**) Sagittal cross-sectional view of the PUF device [27].

Briefly, the fully instrumented PUF integrates both measurement and determination units (Figure 1). The measurement unit features a funnel-shaped housing placed over the urethral meatus to collect urine and includes integrated flow and temperature sensors. This assembly connects to a signal conditioner unit (SCU) worn at the waist. Urine flow rate and temperature are measured using hotwire anemometry principles through a modified version of the flow rate sensor developed by Lin et al. [29], capable of measuring leakage rates from a single droplet to 40 mL/s. The rigid urine channel, with a 10 mm diameter circular cross-section, was rapid-prototyped using a Form 2 3D printer (Formlabs, Somerville, MA, USA). To ensure comfort across various labial shapes, the funnel was overmolded onto the rigid channel using flexible medical-grade silicone elastomer MED2-4220 (Nusil Technology LLC, Carpinteria, CA, USA) [27].

This paper examines the performance of an integrated PUF system with an added flow conditioner (FC) under dynamic conditions that replicate an ADL such as coughing. The system’s efficacy in accurately measuring flow rates over short intervals and across diverse flow rates was assessed. By utilizing computational fluid dynamics (CFD) modeling and machine learning, the study sought to optimize system performance. The goal was to validate the PUF as a reliable tool for continuous monitoring, while addressing the complexities of real-world urinary incontinence scenarios.

## 2. Materials and Methods

### 2.1. Research Design

A simulation-driven conceptual design of a flow conditioner intended for integration into an existing wearable uroflowmeter was developed, aiming to enhance measurement performance under dynamic flow rate conditions. The optimized blade configuration was initially evaluated through benchtop experiments. Additional experimental tests were then conducted to generate a more diverse dataset under varying flow conditions, providing the basis for training and validating machine learning models. These models were employed to analyze experimental data, enabling further refinement of system accuracy and reliability.

### 2.2. Approach

**Figure 2:**
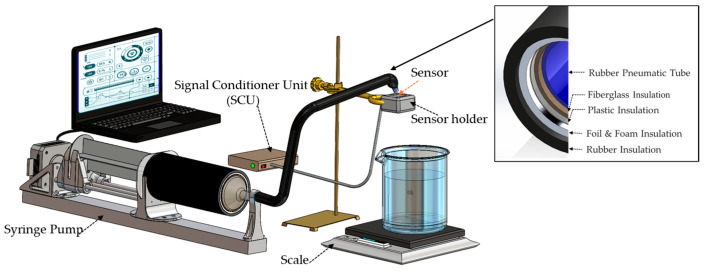
Schematic of the calibration system, featuring a servo-driven syringe pump controlled by a laptop, a thermally insulated syringe and delivery pipe, a PUF sensor with its signal conditioning unit, and a precision scale as the reference measurement (its connection to the laptop not shown for simplicity).

Women with mild-to-moderate incontinence can experience a maximum flow rate during coughing, reaching up to 10 mL in 0.5 milliseconds, which corresponds to a flow rate of 20 mL/s [21,28]. To evaluate our system based on this data, we designed a servo syringe pump system (Figure 2). Briefly, the syringe pump incorporated a commercial 550 mL syringe with a replaced 3D-printed hub designed to decrease flow resistance to high flow rates that was easily connected to a flexible tube with an inner diameter of 9 mm. This inner diameter matched the inner sagittal diameter of the urethra, which has been measured at 8.4 ± 1.9 mm using a vaginal scan and 11.5 ± 2.2 mm when measured using a rectal scan [30]. This servo pump enabled precise control over both flow rate and timing, with a relative standard deviation (RSD) of less than 2.2%.

Figure 2 shows the setup incorporating the syringe pump utilized in the calibration process. The sensor housing was secured in a 3D-printed holder with its urine channel in a vertical orientation, similarly to the urine channel in ADL tests with participants upright and walking or running. Pneumatic air line tubing (OD: 12 mm, ID: 9 mm) was used to transfer water from the syringe to the sensor. A lab stand was used to align the tube above the measurement channel. The fluid output from the measurement channel was directed into a large beaker placed on a high-precision scale (Sartorius ENTRIS6202-1S, Göttingen, Germany), which served as the standard reference. The scale had a resolution of 0.01 gr and was connected to a Lenovo ThinkPad (Lenovo, Morrisville, NC, USA) computer for continuous recording of the instantaneous weight as the beaker filled with water. The PUF sensor was connected to its signal conditioner unit, which recorded the data on a Kingston 32 GB microSD (Kingston Technology Company, Fountain Valley, CA, USA) memory card inside it. The data were uploaded and analyzed after all measurements were completed.

The system underwent optimization and performance enhancement prior to the calibration phase, which involved a series of experimental tests using the setup depicted in Figure 2, simulating human body conditions. To do so, a two-gallon, temperature-controlled water bath (PolyScience, Niles, IL, USA) was used to maintain temperatures between 35 °C and 39 °C. Thermal insulation was applied to the syringe and fluid delivery pipes in the test setup to maintain temperature stability throughout each measurement cycle. As shown in the inset of Figure 2, this insulation included rubber pneumatic pipes, fiberglass, plastic, aluminum foil, foam, and additional rubber layers.

Despite these precautions, the water temperature in the syringe could change when transferred from the water bath. To address this, the water temperature passing through the sensor was measured with a Keysight E2308A thermistor (Keysight Technologies, Santa Rosa, CA, USA) at the beginning and end of each test cycle. The thermistor was housed in a homemade double-walled insulated container filled with bath water to maintain thermal equilibrium, minimizing response time delays and ensuring accurate temperature measurements.

During the optimization phase tests, when the sensor was mounted in its holder, we observed that the sensor did not respond as expected. As will be shown in Section 3 (Results), the coefficient of determination (R^2^) between the flow sensor output and the different flow rates applied to the sensors was only 0.44.

The unsatisfactory response was hypothesized to be due to turbulence in the flow as it entered the measurement channel. In other words, the flow structure significantly influenced measurement accuracy and system performance, particularly when swirl flows and undeveloped velocity profiles were present. For reliable in situ operation, it is known to be essential for flowmeters—typically calibrated under laboratory conditions with fully developed, swirl-free flow—to operate under similar conditions in practice, though practical applications often involve distorted flow conditions [31,32,33].

To test this hypothesis, an open conical channel was designed to maintain laminar flow within the specified flow rate range, with the sensor placed inside. The channel featured a radiused transition from a 7.5 mm to a 2.1 mm diameter. The larger end radius allowed the channel to accommodate higher flow rates, while the smaller end radius provided effective flow control and constriction. The gradual change in radius facilitated smooth flow transitions without inducing turbulence. Under these conditions, the R^2^ value improved significantly to 0.98.

Having confirmed the hypothesis, it became essential to address flow disturbances and reduce turbulence during measurements with the sensor positioned in the measurement channel. Typically, achieving a proper flow velocity profile requires at least 30 L/D of straight piping upstream, where L is the pipe length and D is its diameter [34,35]. However, applying such a long, straight pipe was not practical given the spatial constraints of our measurement channel, which was only 1 cm in length. An alternative approach was to use a flow conditioner combined with a shorter settling length. This device enhanced flow profile development and minimized swirl by homogenizing the velocity distribution, eliminating disturbances, and redistributing the flow into a more stable and uniform pattern [32,33,35].

### 2.3. Flow Conditioner Design and Optimization

Among various FCs recommended in the flow metering standard ISO 5167-1, such as tube bundles, Gallagher, K-Lab NOVA, NEL (Spearman), Zanker flow conditioners, Etoile, AMCA egg box, and Sprenkle devices [32,36], the Etoile FC has been extensively studied for performance enhancements to operate effectively with minimal upstream settling lengths [31,34]. For example, a compact D/8 eight-vane Etoile FC design effectively reduces swirls and enhances flow, as demonstrated by Laws et al. [31,33]. Initially, the proposed short Etoile unit had a central wake caused by a solid blockage at the pipe centerline where the vanes meet [31]. Laws and Ouazzane addressed this by creating an “open” version, removing the central portion [33]. While the standard Etoile design required a longer downstream settling length because of slower wake mixing, the open Etoile unit, with its 0.2D open center, effectively reduced swirl and mitigated upstream flow nonuniformities, improving downstream conditions [31,33]. With this understanding, we used CFD modeling to analyze and compare various FCs, including the Etoile FC. Our results showed that an eight-vane Etoile flow conditioner with a 0.2D opening is the optimal choice for our compact measurement channel.

The CFD model using COMSOL Multiphysics 5.6 software was developed to estimate flow behavior around the sensor and evaluate the performance of FCs during optimization studies. The Turbulent Flow, k-ε interface in COMSOL was used to effectively model and predict turbulent fluid behavior because of its proven accuracy in high-Reynolds-number conditions [37]. In this study, turbulence intensity (TI) along the sensor was employed as a comparative metric to evaluate the performance of various flow conditioner structures. Turbulence intensity was calculated using the relationship $TI=\sqrt{k}/U$ [38], where k denotes the turbulent kinetic energy measured in $m^{2}/s^{2}$ and U is the velocity measured in m/s. Therefore, TI is a nondimensional parameter quantifying the strength of turbulent fluctuations relative to the flow velocity. Furthermore, the FC’s ability to homogenize the velocity profile highlights the importance of comparing time-averaged axial velocity components along the downstream horizontal line at the sensor position for different design structures.

**Figure 3:**
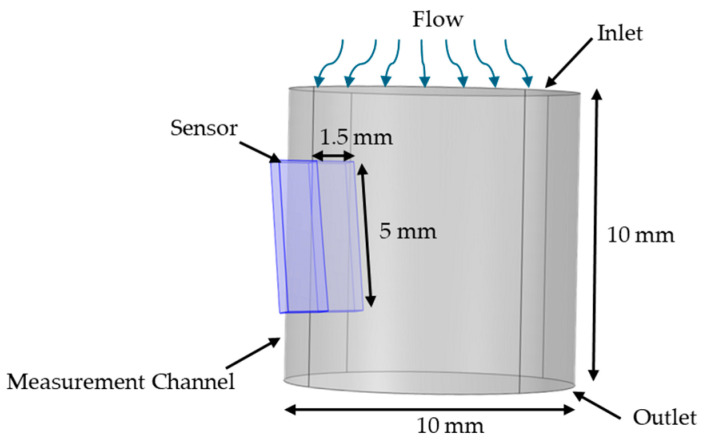
CFD model of the sensor in the measurement channel, with arrows showing the flow direction.

The CFD model of the measurement channel is shown in Figure 3. The sensor’s glass substrate was represented as a rectangular prism within the measurement channel, with the software modeling only the fluid region and excluding the parts of the prism outside the channel. The attack angle shown in the model was included because experimental evidence indicated that a small sensor attack angle was critical to prevent fluid separation on the side of the sensor die where the filaments are deposited. Without this inclination, eddy currents formed over the filament, thereby increasing turbulence and leading to inconsistent results. The upper side of the measurement channel served as the inlet, and the lower side was designated as the outlet.

**Figure 4:**
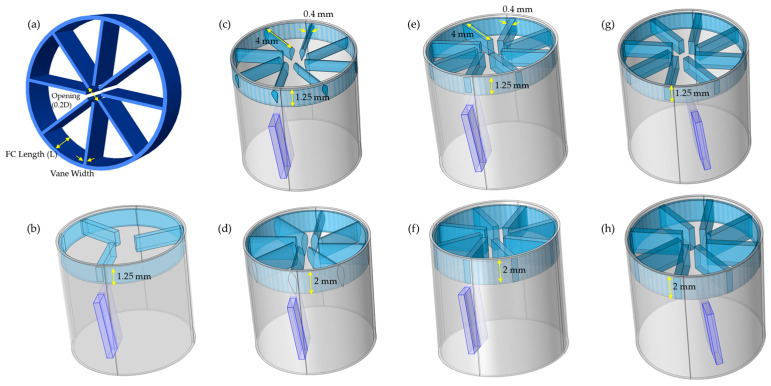
(**a**) Schematic of the flow conditioner design and its location relative to the sensor within the measurement channel. Illustration of different FC designs: (**b**) 3-vane Etoile FC, (**c**) airfoil-shaped 8-vane FC, (**d**) extended airfoil-shaped 8-vane FC, (**e**) classic Etoile configuration, (**f**) extended classic Etoile configuration, (**g**) sensor positioned between vanes in structure (**e**), and (**h**) sensor positioned between vanes in structure (**f**).

We then integrated the FC into the measurement channel. Based on the FC design proposed by Laws et al. [31], for a measurement channel diameter of 1 cm, the optimal flow conditioner length was set at D/8 = 1.25 mm, with a 2 mm opening. The vane width, constrained by the capabilities of the Form 2 3D printer (Formlabs, Somerville, MA, USA), was chosen at 40 µm, the minimum achievable with this printer. The structure of this design is illustrated in Figure 4a.

In the next phase, we refined the design for seamless integration and optimal performance within our system. This involved conducting optimization studies to determine the best vane shape, count, width, flow conditioner length, and sensor positioning relative to the FC’s vanes (Figure 4. Given the limited size of our measurement channel and the benefits of reduced vane count to minimize flow obstruction and turbulence, we compared eight-vane and three-vane Etoile configurations (Figure 4b,e). To mitigate the flow separation and turbulence caused by sharp-edged vanes, we explored airfoil-shaped vanes designed for smoother aerodynamics (Figure 4c). After identifying the upstream eight-vane Etoile as the most effective configuration, we further refined it by increasing the height to 2 mm (Figure 4d,f) to ensure sufficient settling lengths for optimal flow stabilization. This adjustment represented the maximum permissible height above the sensor within the measurement channel. We also tested the sensor’s positioning relative to the vanes—either directly beneath a vane or positioned between two vanes (Figure 4g,h). Then, the turbulence intensity and velocity profile along the sensor for all configurations were analyzed.

**Figure 5:**
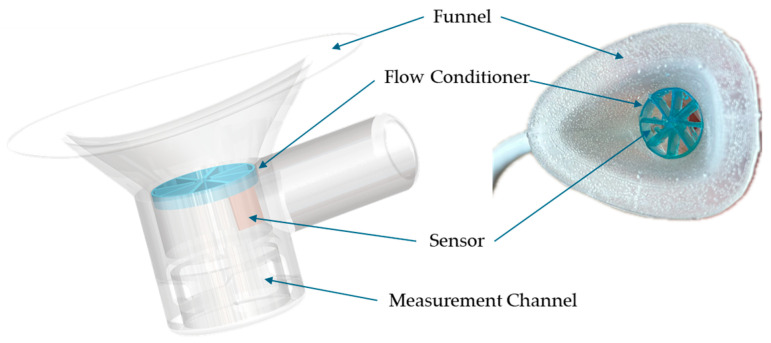
Placement of the 3D-printed flow conditioner within the PUF measurement channel.

The selected design was fabricated using a Form 2 3D printer. To address potential 3D printing errors, multiple parts were printed to ensure precise dimensional accuracy. The flow conditioner was affixed upstream of the sensor within the measurement channel (Figure 5). The literature suggests that positioning flow conditioners closer to the disturbance source can enhance their effectiveness in mitigating flow disturbances [38]. Because of the limited space available upstream of the sensor in our compact measurement channel, the flow conditioner was placed at the entry point of the channel. This positioning was also considered in the CFD model.

### 2.4. Data Collection and Processing

#### 2.4.1. Dataset and Preprocessing

Data were collected over nine measurement cycles, with each cycle employing 14 distinct flow rates ranging from 0 to 20 mL/s. Each flow rate was applied for a duration between 2 and 6 s, with a one-minute interval between successive flow rates. The sensors’ outputs, along with corresponding timestamps, were recorded onto the memory card. Post-experiment, the data were extracted and processed by converting sensor readings to millivolts and timestamps to seconds. Each measurement cycle was then segmented to identify tests conducted at different flow rates.

The PUF’s sensors’ die contained two filaments, each identically designed and sensitive to both the temperature and flow rate of the fluid passing over them. By operating at different input power levels, one filament primarily responds to flow rate while the other is more sensitive to temperature [27,29]. By leveraging the outputs from these two filaments, both flow rate and temperature can be measured. Despite the intended design, the temperature-sensitive filament exhibited some sensitivity to flow rate. Given the concurrent variability in temperature and flow rate during measurements, feature extraction from the temperature sensor output is challenging. To address this, we subtracted the outputs of the two sensors—after applying min–max normalization to compensate for differences in the sensors’ gain—to yield a single, combined output. In addition, the true flow rate and temperature values were obtained from the syringe pump system and the Keysight thermostat, respectively. Consequently, the dataset comprised 126 measurements, with each entry including the flow rate sensor output (FSO), the flow rate–temperature differential (FTD), timestamps (t), and the corresponding true flow rate (TFR) and true temperature (TT).

Machine learning methods were then employed to analyze the data and evaluate the system’s performance. The ML algorithm was expected to model the relationship between sensor outputs and the corresponding flow rate.

#### 2.4.2. Feature Selection and Transformation

Data normalization/standardization is a critical preprocessing step to enhance ML model performance [39]. Among the different methods evaluated, Z-score standardization was chosen for its effectiveness in achieving comparable feature distributions. This method standardizes each feature by centering it around a mean of zero and scaling to unit variance, ensuring that features with different scales (such as FSO and FTD) contribute equitably without disproportionately affecting the results.

Selecting the right features is a fundamental aspect of ML. By reducing dimensionality and concentrating on the most pertinent variables, feature selection helps decrease error rates and improve the overall effectiveness of models [40,41]. Although feature reduction was not our primary focus because of the limited number of inputs, we explored the potential benefits of combining FSO and FTD, given their sensitivities to temperature and flow rate, respectively. One approach considered was combining FSO and FTD into a single metric (FTS) to better capture their relationship with TFR. To mitigate multicollinearity and extract the most informative components, we applied principal component analysis (PCA), which transforms correlated variables into uncorrelated principal components [40,42]. Additionally, kernel PCA was evaluated to capture potential nonlinear relationships when the data were not linearly separable [43].

To quantify associations among these variables, Spearman correlation analysis was performed, highlighting their links to TFR. Based on these insights, correlation-based feature selection (CFS) was considered to identify feature subsets with strong correlations to the target variables [39]. Finally, we evaluated machine learning models’ performance using different feature subsets.

#### 2.4.3. Machine Learning Models

We focused on widely used machine learning models relevant to biosensors, prioritizing regression-based supervised methods with low computational costs. Given their proven effectiveness in processing wearable biosensor data, we considered four models: support vector machine (SVM), random forest (RF), extreme gradient boosting (XGBoost), and neural network (NN) [39,44,45,46]. RF and XGBoost are ensemble methods that leverage decision trees, while SVMs use kernel functions to effectively handle nonlinear relationships. Additionally, NNs use layered neurons and backpropagation to learn complex patterns and nonlinear relationships [44].

Random forest improves accuracy and reduces overfitting by constructing an ensemble of decision trees trained on random data subspaces. It measures feature importance through impurity decrease, is robust to outliers, and captures nonlinear relationships with minimal feature engineering. Its use of bagging and random feature selection mitigates overfitting, while out-of-bag error estimation aids in optimizing model parameters [39,40,41,44,45,47,48]. For instance, ref. [49] highlights the use of RF to refine feature selection and regression in an inkjet-printed, body-worn temperature sensor, demonstrating its potential to advance wearable technologies for physiological monitoring.

SVM finds the optimal hyperplane for regression by using kernel functions, which map data into higher-dimensional spaces when linear separation is not possible. Its effectiveness relies on selecting the appropriate kernel type and parameters based on the data’s characteristics. The dual problem formulation in SVM allows efficient handling of constraints and enables kernel functions to capture complex decision boundaries [40,47]. For example, ref. [50] utilized SVM to estimate blood glucose levels from breath samples, choosing SVM for its adaptability and minimal memory requirements, which rendered it ideal for developing a scalable, portable biosensor.

Ensemble methods such as XGBoost combine the strengths of multiple learners to enhance prediction accuracy [40]. This approach, based on gradient boosting machines, iteratively reduces loss by adding weak learners and evaluating their contributions to the features [39,45]. It reduces overfitting through regularization, early stopping, and sequential tree corrections. XGBoost captures nonlinear relationships with minimal feature engineering and measures feature importance using gain, cover, and weight metrics [51]. In [52], XGBoost was applied to analyze data from wearable sensors embedded in shoes, enhancing the prediction of fall risk levels in older adults. The algorithm improved the prediction’s accuracy by effectively processing complex gait data, showcasing its utility in advanced health monitoring systems.

Thus, each of these models—SVM, XGBoost, and RF—has its own advantages and limitations in biosensor applications. In this study, these models were developed and trained using the training dataset to evaluate their performance.

NNs are also widely used in biosensor applications, such as [53], where an NN enhanced the accuracy of identifying characteristic points in wearable ECG signals despite challenges such as high noise. However, small datasets can limit ML model performance, particularly for NNs, because of overfitting risks. Typically, SVMs perform better on limited data, followed by RF, whereas NNs require extensive tuning [54,55]. To address these constraints, we utilized AutoGluon’s AutoML framework to optimize and integrate the NN effectively. AutoGluon automates hyperparameter tuning, model selection, and ensembling, employing regularization techniques (e.g., dropout) to reduce overfitting and variance through ensemble learning, thus enhancing generalization and performance [56]. By automating these processes, AutoGluon allowed us to integrate NNs with SVM, RF, and XGBoost, leveraging comprehensive optimization capabilities even in limited-data scenarios.

#### 2.4.4. Hyperparameter Optimization

Hyperparameters for each model were tuned via an exhaustive grid search. In RF, parameters such as the number of trees, minimum samples for splits and leaves, and the bootstrap setting were optimized. Adjusting these parameters helps balance overfitting and underfitting while improving generalization [35,41,43]. In SVM models, hyperparameters such as the regularization parameter C, kernel coefficient gamma, and kernel type were optimized, while for XGBoost, the learning rate, maximum tree depth, and fraction of features used for each tree were fine-tuned to optimize model performance and prevent overfitting, ensuring the best possible balance between bias and variance. Using AutoGluon, model performance was further refined through automated hyperparameter tuning, focusing on reducing the mean square error (MSE). This streamlined the integration of the NN, XGBoost, SVM, and RF models, enhancing accuracy while minimizing manual effort intervention.

The hyperparameters and their corresponding values used during GridSearchCV are presented in Table 1. These parameters were selected based on their default settings and a range designed to encompass the expected variability in model performance. In AutoGluon, we employed similar parameters for RF, SVM, and XGBoost.

#### 2.4.5. Evaluation Matrices

For performance evaluation, the dataset was split into training (80%) and testing (20%) sets using random stratified sampling. Cross-validation (10-fold) was applied exclusively to the training set via GridSearchCV to optimize hyperparameters. Performance metrics such as R-squared, mean absolute error (MAE), and root mean square error (RMSE) were used to assess model performance, where R-squared quantifies explained variability, MAE measures the average prediction error, and RMSE penalizes larger errors more significantly.

## 3. Results

**Figure 6:**
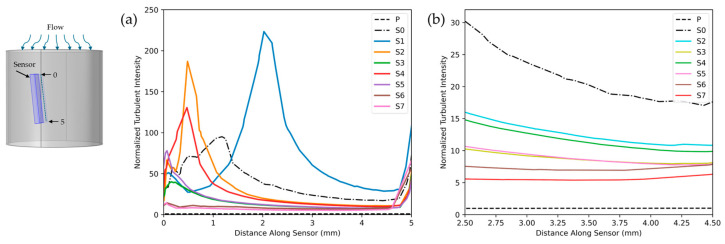
(**a**) Normalized turbulence intensity along the sensor for different flow conditioner designs; (**b**) zoomed-in view of (**a**) from 2.5 to 4.5 for enhanced comparison. Source data and analyses available; see Data Availability Statement.

Figure 6a compares the turbulence intensity along the sensor position for various FC designs, with a zoomed-in view for enhanced comparison Figure 6b. **P** represents the measurement channel without the sensor with laminar flow, while **S0** refers to the measurement channel with a sensor under turbulent flow conditions. **S1** used the configuration in Figure 4b, featuring a D/8-length, three-vane Etoile FC with a 0.2D opening. **S2** used a D/8-length, eight-vane, airfoil-shaped FC with a 0.2D opening (Figure 4c), and **S3** extended this design by increasing its length to 2 mm (Figure 4d). Similarly, **S4** followed the configuration in Figure 4e, incorporating a D/8-length, eight-vane Etoile FC with a 0.2D opening, while **S5** was built on **S4** by extending the FC length to 2 mm (Figure 4f). In **S6**, the structure of **S4** was modified by repositioning the sensor between the vanes (Figure 4g), whereas **S7** followed the same approach as **S6** but applied it to **S5**, placing the sensor between the vanes (Figure 4h). For ease of comparison, all results were normalized against the TI value of laminar flow in the measurement channel (**P**).

**Figure 7:**
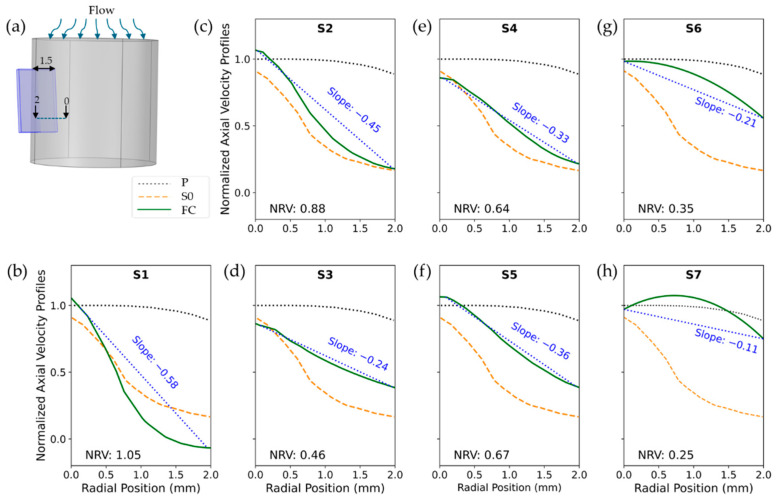
Time-averaged axial velocity profiles at the sensor location shown in (**a**) for S1 (**b**), S2 (**c**), S3 (**d**), S4 (**e**), S5 (**f**), S6 (**g**), and S7 (**h**) compared with P and S0. The dotted black line represents P, the dashed orange line corresponds to S0, and the solid green lines depict various FC structures. Source data and analyses available; see Data Availability Statement.

Figure 7 illustrates the performance of various flow conditioner designs in homogenizing velocity profiles. The figure shows normalized, time-averaged axial velocity profiles measured at the sensor location downstream (Figure 7a). Each curve contrasts a flow conditioner’s profile with two benchmarks: (1) the ideal laminar profile in the measurement channel (P) and (2) the turbulent profile in the sensor-equipped channel (S0). All velocities were normalized by the maximum laminar flow velocity in laminar flow.

For enhanced comparison of different FC structures with respect to velocity homogeneity, we illustrate the slope of the line connecting the initial and final points of each velocity profile. Additionally, to capture the overall variation—particularly in structures such as S6 and S7, where the velocity increased from the starting point and then decreased—we report the normalized range of velocity (NRV). The NRV was defined as the difference between the minimum and maximum velocities within the profile, normalized by the corresponding laminar flow velocity in the pipe, and is presented in each figure.

**Figure 8:**
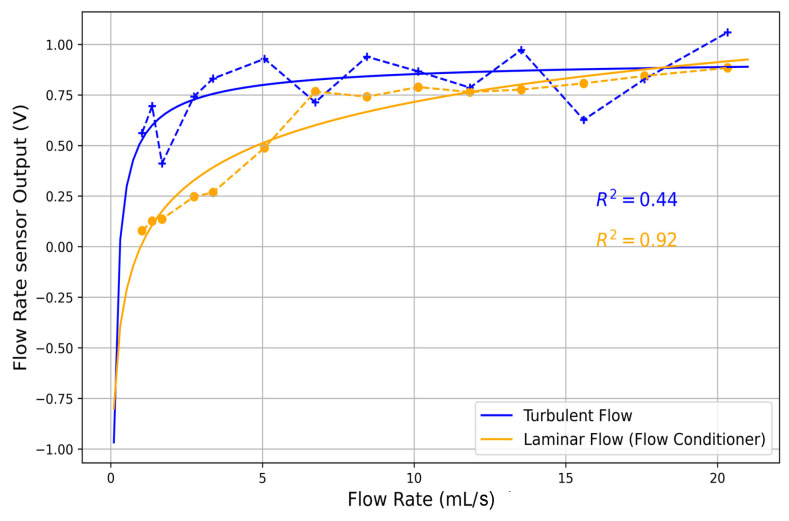
Flow sensor output under turbulent (blue) and laminar (orange) flow conditions. Each point represents the mean of five measurements, connected by a dashed line, with the solid line indicating the fitted curve. Source data and analyses available; see Data Availability Statement.

Following the above results, the eight-vane Etoile flow conditioner was chosen, 3D-printed, and installed in the measurement channel. Figure 8 presents experimental results, comparing the flow sensor output with and without FC across fluid flow rates ranging from 0 to 20 mL/s. Each point represents the mean of five measurements, with the solid line indicating the fitted curve.

**Figure 9:**
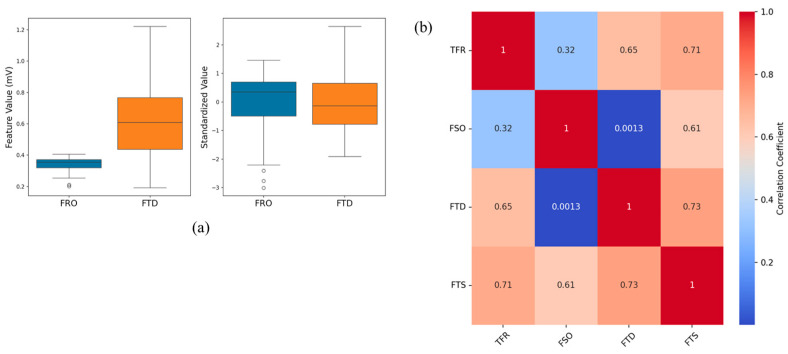
(**a**) Z-score standardization of FSO and FTD; (**b**) correlation analysis between inputs and TFR. Source data and analyses available; see Data Availability Statement.

In our analysis, we first applied Z-score standardization, with Figure 9a showing its effect in ensuring that FSO and FTD contributed equally to the ML models. Figure 9b presents a correlation matrix detailing the relationships between the inputs and the output, providing a deeper insight into the interdependencies among these variables. This analysis revealed not only the direct relationships, such as the strong correlation of the composite metric FTS with TFR, but how the individual features contributed to the overall prediction.

Based on these insights, we evaluated multiple models—RF, SVM, and XGBoost, with AutoGluon used for automated tuning and incorporating NNs—to determine which algorithm could best predict the flow rate from the sensor outputs. Table 1 presents the selected hyperparameters for each model among the values provided for GridSearchCV across each feature subset, including **T1**: FTS; **T2**: FTD; **T3**: FSO, FTS, and FTD; **T4**: FSO, FTS, and FTD with PCA (linear); and **T5**: FSO, FTS, and FTD with PCA (RBF). The table also includes the time cost for each model, incorporating grid search durations, allowing for a comparison of training times across models.

**Table 1:** Value ranges explored during GridSearchCV, along with the selected hyperparameters and total training times (including grid search), for each feature subset (T1–T5). Source data and analyses available; see Data Availability Statement.

Model	Hyperparameter	Values	Best Parameters					Time Cost
			T1	T2	T3	T4	T5	
RF	n_estimators	30, 40, 50, 100	30	40	30	50	50	88.8 s
	max_depth	10, 20, 30, 40	30	40	30	30	30	
	min_samples_split	2, 5, 10	2	10	2	5	2	
	min_samples_leaf	1, 2, 4	4	4	2	1	1	
	bootstrap	True, False	True	True	True	True	True	
XGBoost	n_estimators	40, 50, 100, 200	50	40	40	50	50	16 s
	learning_rate	0.01, 0.05, 0.1, 0.2	0.05	0.05	0.1	0.1	0.1	
	max_depth	3, 5, 7, 10	3	3	10	3	3	
	subsample	0.6, 0.8, 1.0	1.0	0.8	1.0	0.6	0.6	
	colsample_bytree	0.6, 0.8, 1.0	0.6	0.6	0.8	1.0	1	
SVM	C	0.1, 1, 10, 100	10	1	100			2 s
	Gamma	‘scale’, ‘auto’, 0.1, 0.01, 0.001	Scale	Scale	Scale			
	kernel	‘linear’, ‘rbf’, ‘poly’, ‘sigmoid’	rbf	rbf	rbf			
NN in AutoGloun	num_layers	1, 2, 3, 4						9.1 s *
	dropout_prob	0.1, 0.2, 0.3, 0.4						

* This time cost is for the AutoGluon model, which includes RF, SVM, XGBoost, and NN.

**Figure 10:**
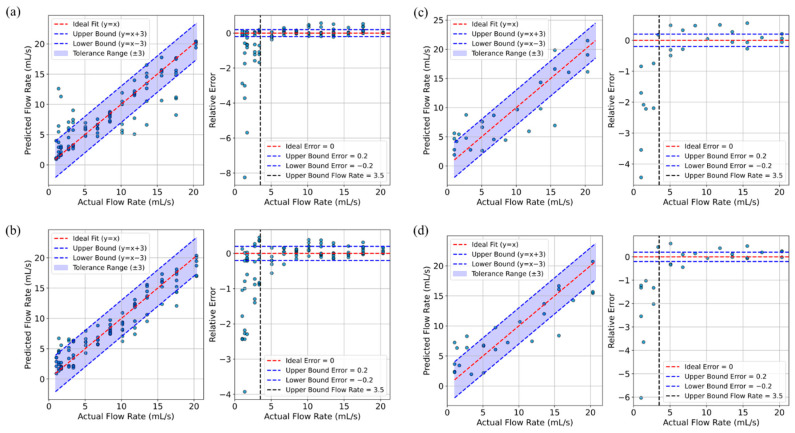
Predicted vs. actual flow rates and absolute error for SVM (**a**,**c**) and XGBoost (**b**,**d**) models on training (**a**,**b**) and test data (**c**,**d**). Source data and analyses available; see Data Availability Statement.

Finally, Table 2 summarizes the performance metrics for these models with optimized hyperparameters, including the correlation between TFR and the predicted flow rate and R^2^ values for both training and test sets, as well as the MAE and RMSE for the test data. These results were examined across various input combinations and with or without the application of kernel PCA. Ultimately, the best performance was achieved by the SVM model using FSO, FTD, and FTS as inputs and the XGBoost model with linear PCA applied to the same inputs. Figure 10 further illustrates the relationship between the predicted flow rates and the true flow rate, alongside the corresponding relative errors across different flow rates, for these two selected models.

## 4. Discussion

This study presents the process we used for improving the performance of the PUF under dynamic conditions, so as to more accurately monitor urinary incontinence in ADLs. Our approach involved hardware innovations—namely, the sensor housing design—along with a comprehensive analysis of the fluid dynamics using computational fluid dynamics and experimental validation, followed by the application of machine learning for flow rate prediction.

### 4.1. Sensor Performance and Flow Dynamics

Initial tests of the PUF in a dynamic environment revealed a significant challenge. As shown in Figure 8 the flow rate measurements had low accuracy (R^2^ = 0.44) due to turbulence. To address this, we tested the sensor in a conical channel to induce laminar flow, improving the R^2^ to 0.98. This confirms the importance of laminar flow for accurate sensor readings.

To achieve a more uniform flow profile in the short measurement channel, we incorporated the FC, starting with the eight-vane Etoile design, with a vane opening of 0.2D and length D/8. Our CFD simulations allowed us to compare various vane structures by analyzing normalized turbulence intensity and axial velocity profile at the sensor location.

Flow TI quantifies the magnitude of velocity fluctuations, with lower TI values ensuring more stable and accurate downstream measurements. Similarly, the axial velocity profile provides insight into flow stability and uniformity along the streamwise direction, where a lower slope and smaller NRV indicate improved consistency.

Our analysis in Figure 7 showed that the S1 configuration, with only three vanes, performed poorly, exhibiting the highest TI peak along with a steep velocity profile slope of −0.58 and an NRV of 1.05. This was likely due to the larger gaps between vanes, which allowed turbulence to persist rather than being effectively redistributed. In contrast, the denser eight-vane arrangement acted as a more effective FC, disrupting turbulence and promoting a more uniform velocity profile with reduced TI.

Comparing FC lengths revealed that increasing the FC length from 1.25 mm to 2 mm resulted in a significant TI reduction—42% for S3 versus S2 and 35% for S5 versus S4. This improvement was likely due to the extended length providing additional space for turbulence dissipation and flow reorganization, reducing the influence of upstream disturbances. As a result, the vane shape becomes less critical. Notably, at an FC length of 2 mm, both the airfoil-shaped (S3) and rectangular (S5) vanes yielded similar TI values, suggesting that the specific vane geometry had a negligible effect on turbulence under these conditions. However, the airfoil configuration produced a more uniform velocity profile, with a slope of −0.24 and an NRV of 0.46.

**Figure 11:**
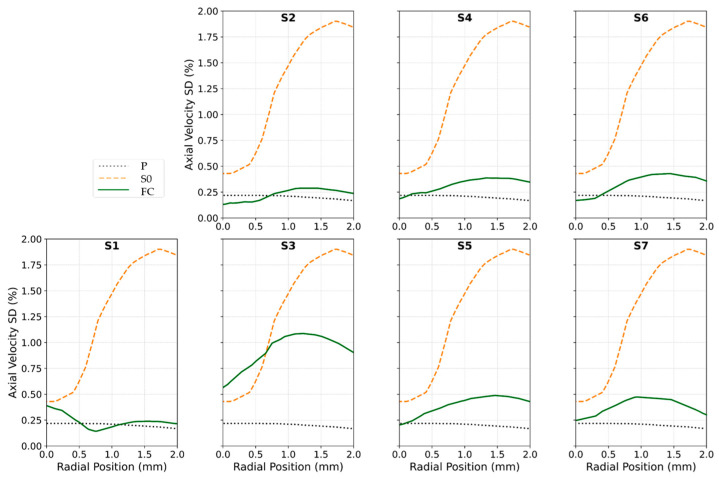
Standard deviation of the axial velocity across spatial locations for S1 to S7 compared with P and S0. The dotted black line represents P, the dashed orange line corresponds to S0, and the solid green lines depict various FC structures. Source data and analyses available; see Data Availability Statement.

To better differentiate between these two vane shapes, we also evaluated the axial velocity standard deviation (SD) as a measure of temporal stability. Higher SD values indicate stronger unsteady fluctuations, whereas lower values suggest a more stabilized flow. As shown in Figure 11, the axial velocity SD for S3 ranged from 0.6% to 1.1%, while for S5, it was significantly lower, ranging from 0.2% to 0.4%. This suggests that the rectangular vane (S5) produced a steadier flow, making it the better choice. The higher fluctuations in S3 likely stemmed from the airfoil’s curved geometry, which enhanced shear layer formation and delayed wake stabilization.

Building on these findings, we further refined the S5 configuration by testing different sensor positions. The simulations indicated that positioning the sensor between the vanes decreased the measured TI by 65% (S6 vs. S4) and 60% (S7 vs. S5). This reduction was attributed to placing the sensor in a more stabilized flow region, away from high-shear and recirculation zones. Among all configurations, S7 demonstrated the best performance, achieving an 82% reduction in TI compared to S0. It also exhibited the smallest velocity profile slope (−0.11), the lowest NRV (0.25), which was 67% lower than that of S0, and a reduction in velocity SD from 0.47% to 0.25%.

### 4.2. Experimental Integration and Calibration

The integration of the optimized FC into the measurement channel led to a significant improvement in sensor performance. While the FC affected local flow velocity over the sensor, this impact was manageable through system calibration to compensate for the shift.

The device was at this point ready for calibration, considering variable body temperatures and dynamic conditions to ensure accurate sensor output correlation with true flow rates.

### 4.3. Machine Learning Model Development

To address residual turbulence, temperature variations, and the sensor’s nonlinear behavior, we developed machine learning models predicting TFR using sensor outputs FSO, FTD, and their composite metric FTS. The correlation table (Figure 9b) highlights key insights, notably the strong correlation of FTS with TFR (0.71), making it an effective predictor. FSO and FTD also showed high correlations with both FTS and TFR (FTD correlating 0.65 with TFR and 0.73 with FTS), indicating that each input captured distinct aspects of flow dynamics. This underscores the benefits of a multifeature approach, which enhances predictive performance by providing complementary information. The presence of high correlations among inputs did not hinder our analysis, as tree-based models such as RF and XGBoost inherently manage multicollinearity by emphasizing feature importance. PCA was used to reduce dimensionality and mitigate potential issues from correlated inputs. We also evaluated the SVM model, which effectively handles correlated variables, ensuring that our multifeature strategy remained robust.

Based on comparative analyses (see Table 2), we prioritized models that demonstrated strong performance on test data and maintained consistency between training and test results. Significant performance gaps would indicate overfitting and a lack of consistency across the dataset. For instance, while AutoGluon using FTD as input provided an R^2^ of 0.75 on test data, the training R^2^ was only 0.35.

Ultimately, the best-performing models were the SVM and the XGBoost-PCA with a linear kernel. The SVM achieved a training correlation of 0.88 and an R^2^ of 0.78, while its test performance showed a correlation of 0.86, R^2^ of 0.74, MAE of 2.8, and RMSE of 3.4. In comparison, the XGBoost-PCA linear model delivered even stronger training performance, with a correlation of 0.95 and an R^2^ of 0.9, and comparable test metrics—a correlation of 0.88, R^2^ of 0.76, MAE of 2.6, and a lower RMSE of 3.3. As illustrated in Figure 10, the slight advantage of the XGBoost-PCA model suggests it may provide more accurate flow rate predictions. Although RF-PCA produced results comparable to those of XGBoost-PCA, we ultimately selected XGBoost-PCA for its slightly superior performance.

These findings highlight the advantage of combining FTS, FSO, and FTD, as each contributes unique, complementary insights into flow dynamics, resulting in more robust and reliable predictions—essential for navigating the complexities of the dynamic measurement environment in our wearable uroflowmeter. Additionally, our results indicate that relative error was higher for flow rates below 3.5 mL/s, likely because of incomplete sensor coverage at low flow rates.

As the training costs for each model are presented in Table 1, it is important to highlight that these times included grid search durations. This aspect may have contributed to variations in time costs across models due to differences in the number of hyperparameter values evaluated in addition to the characteristics of the models themselves. Given our small dataset, the models with optimized hyperparameters ran swiftly.

With a carefully designed flow conditioner integrated into the measurement channel and the application of suitable machine learning models with optimized hyperparameters and optimal feature selection, the PUF system is ready for dynamic situations similar to ADLs. Overall, these findings pave the way for future advancements in monitoring technologies, offering promising solutions for improving efficiency and accuracy in practical applications.

## 5. Conclusions

In conclusion, this research demonstrates significant advancements in the functionality of a wearable flowmeter intended for monitoring urinary incontinence in dynamic conditions. By integrating a specifically optimized eight-vane Etoile flow conditioner featuring a 0.2D opening, the flowmeter markedly improved the stability and accuracy of flow measurements. This enhancement led to an increase in the R^2^ value of our measurements from 0.44 to an impressive 0.92. Moreover, the utilization of machine learning techniques achieved an R^2^ of 0.76 on test datasets, highlighting the strong potential of our system for practical applications. The combined use of CFD simulations, experimental validation, and machine learning modeling provides a robust framework to tackle the challenges of turbulent flow in real-time measurement scenarios. Looking forward, we plan to expand our dataset to enhance the generalizability and performance of our model across diverse conditions, ensuring its robustness and applicability in various situations. Furthermore, future research involving human subjects is being considered to comprehensively validate the effectiveness of our system and its machine learning models. These developments mark critical progress toward creating reliable monitoring devices that can significantly aid individuals managing urinary incontinence.

## Figures and Tables

**Table 2:** Comparison of the performance of the different machine learning models in predicting flow rate for training and test data. Source data and analyses available; see Data Availability Statement.

			Training Data		Test Data			
Input	PCA	ML Model	Corr	R^2^	Corr	R^2^	MAE	RMSE
T1: FTS	-	RF	0.84	0.7	0.8	0.63	3.3	4.1
		SVM	0.75	0.54	0.77	0.58	3.5	4.3
		XGBoost	0.85	0.7	0.77	0.55	3.7	4.5
		AutoGluon	0.85	0.69	0.8	0.57	3.8	4.4
T2: FTD	-	RF	0.74	0.54	0.84	0.65	3.2	4
		SVM	0.67	0.44	0.84	0.69	3	4
		XGBoost	0.81	0.63	0.83	0.63	3.5	4.1
		AutoGluon	0.66	0.35	0.87	0.75	2.5	3.4
T3: FSO FTS FTD	-	RF	0.96	0.91	0.86	0.73	2.7	3.5
		SVM	0.88	0.78	0.86	0.74	2.8	3.4
		XGBoost	1	1	0.84	0.68	3	3.8
		AutoGluon	0.88	0.76	0.84	0.7	2.9	3.7
T4: FSO FTS FTD	Linear	RF	0.97	0.94	0.87	0.75	2.8	3.4
		XGBoost	0.95	0.9	0.88	0.76	2.6	3.3
T5: FSO FTS FTD	rbf	RF	0.93	0.85	0.78	0.6	3.3	4.2
		XGBoost	0.94	0.87	0.81	0.65	3.2	4

## Data Availability

The datasets can be found at https://doi.org/10.7302/1zth-8c11.

## Abbreviations

The following abbreviations are used in this manuscript:

ADL	Activities of daily living
CFD	Computational fluid dynamics
FC	Flow conditioner
FSO	Flow-rate sensor output
FTD	Differential of flow-rate and temperature sensors
FTS	Composite metric of FSO and FTD
MAE	Mean absolute error
MSE	Mean squared error
NN	Neural network
NRV	Normalized range of velocity
PCA	Principal component analysis
PUF	Personal uroflowmeter
RF	Random forest
RMSE	Root mean square error
SCU	Signal conditioner unit
SVM	Support vector machine
TFR	True flow rate
TI	Turbulence intensity
TT	True temperature
UI	Urinary incontinence
XGBoost	Extreme gradient boosting
